# Wearable Devices for Remote Monitoring of Heart Rate and Heart Rate Variability—What We Know and What Is Coming

**DOI:** 10.3390/s22228903

**Published:** 2022-11-17

**Authors:** Navya Alugubelli, Hussam Abuissa, Attila Roka

**Affiliations:** Division of Cardiology, Creighton University and CHI Health, 7500 Mercy Rd, Omaha, NE 68124, USA

**Keywords:** heart rate, heart rate variability, cardiovascular risk, remote monitoring

## Abstract

Heart rate at rest and exercise may predict cardiovascular risk. Heart rate variability is a measure of variation in time between each heartbeat, representing the balance between the parasympathetic and sympathetic nervous system and may predict adverse cardiovascular events. With advances in technology and increasing commercial interest, the scope of remote monitoring health systems has expanded. In this review, we discuss the concepts behind cardiac signal generation and recording, wearable devices, pros and cons focusing on accuracy, ease of application of commercial and medical grade diagnostic devices, which showed promising results in terms of reliability and value. Incorporation of artificial intelligence and cloud based remote monitoring have been evolving to facilitate timely data processing, improve patient convenience and ensure data security.

## 1. Introduction

Heart rate (HR) and heart rate variability (HRV) are important physiologic markers of homeostasis and may provide an early warning for certain abnormal conditions. Advances in technology made ambulatory monitoring of HR and HRV feasible—initially, in the medical field and more recently, using consumer grade devices in the general population. Cardiovascular medicine—especially cardiac electrophysiology—has been an important driver in the evolution and utilization of wearable devices.

HRV has been extensively studied in clinical studies in various applications. The ease of ambulatory heart rate measurement using consumer grade technology increased the interest of HR/HRV monitoring even in non-clinical applications. Advances in analytic methods (linear, non-linear and most recently, machine learning) have the promise to extract more information from the gathered data. As the utilization of these devices continues to increase, it is important to understand the physiological and technical aspects, together with the evidence supporting the uses of wearable technology for specific indications. Recent reviews provide an overview on currently available devices and their proposed utility [1,2]. By reviewing the evidence behind specific aspects of ambulatory HR/HRV monitoring using wearable devices, we can get a better understanding on the appropriate use of this novel technology in this field.

In this review, we will discuss the concepts behind cardiac signal generation, factors affecting HRV, methods of recording, processing and analysis. After a historical perspective, data supporting the use of consumer grade devices will be reviewed, with current issues and future opportunities.

## 2. Electrical Cardiac Signal Generation, Recording

The heart is a complex multichambered pump with continuous mechanical and electrical activity. The ease of measurement of the electrical activity and its strong correlation with mechanical and metabolic activity of the heart made electrocardiography (ECG) one of the most frequently performed non-invasive diagnostic tests.

Cardiac activity generates a continuous electrical signal that must be recorded via electrodes, then filtered and digitalized for analysis. For each cycle of electrical cardiac activity, multiple signals can be seen on the body surface electrocardiogram—in normal sinus rhythm, these are usually a P wave (atrial depolarization), QRS complex (ventricular depolarization) and T wave (ventricular depolarization). The QRS complex represents the onset of ventricular contraction (systole), has the largest amplitude and easiest to detect. Most clinical and commercial ECG-based heart rate monitors use algorithms to identify the QRS complex on the electrocardiogram, then transform it to a series of intervals (RR intervals), which are used to measure HR and changes of HR over time (HRV). In practice, single channel recording is adequate to assess HR/HRV. For more complex analysis, such as arrhythmia morphology or ischemia, multi-channel ECG recordings are utilized.

**Figure 1:**
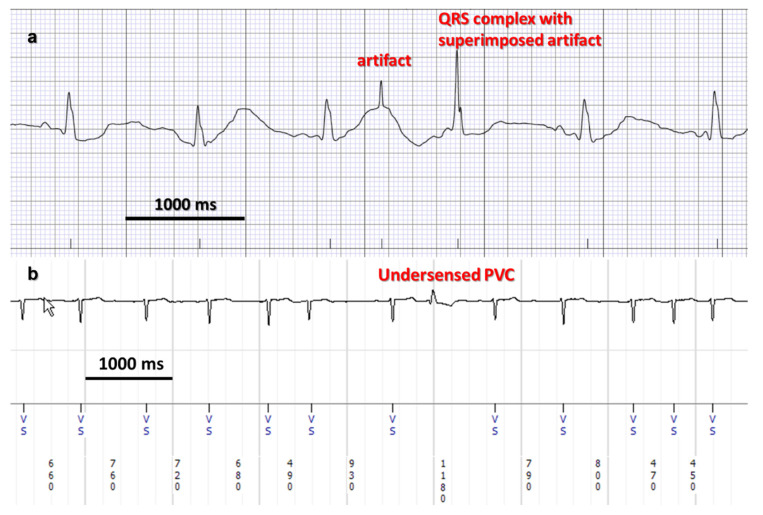
Electrocardiographic recordings of heart activity. (**a**) consumer grade heart rate monitor (AliveCor Kardia). The algorithm detects the QRS intervals and transforms them into markers. An inappropriately marked artifact is seen (non-physiological signal, no repolarization). These findings are easily detected and rejected by trained human interpreters but remain challenging for automated rhythm analysis. (**b**) Implantable cardiac rhythm monitor (Medtronic Reveal Linq). These devices benefit from improved signal/noise ratio due to the subcutaneous position of the device, close to the heart. However, sensing issues are still not infrequent, such as in this patient with atrial fibrillation and an undersensed premature ventricular contraction (PVC).

Proper detection of QRS complexes is of paramount importance to avoid registering signals not related to ventricular activity—this affects both HR and HRV calculations. Due to wide range of cardiac pathology and various environmental factors, the potential for misinterpretation of artifacts remains high and ECGs are overread by trained practitioners in clinical practice (Figure 1).

**Figure 2:**
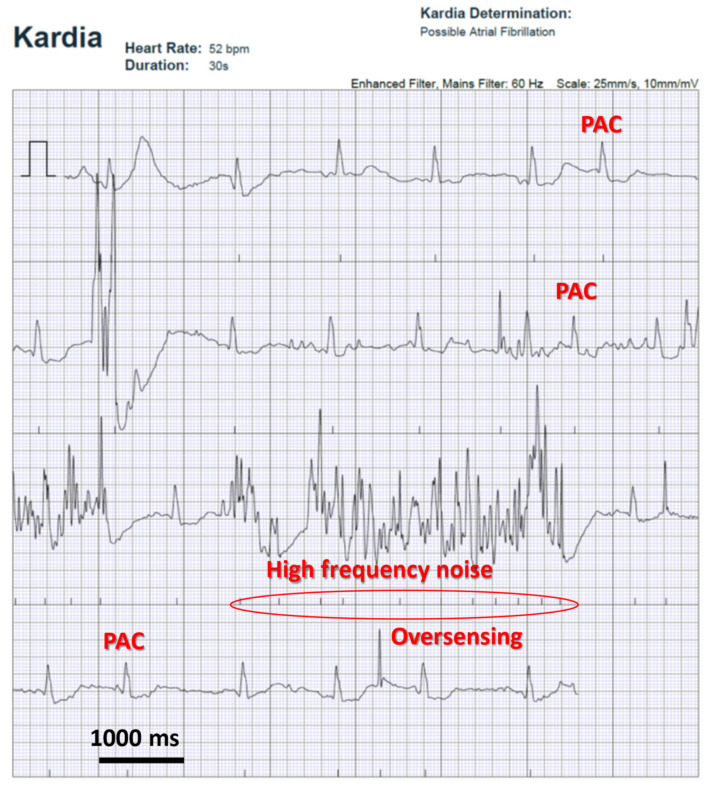
Electrocardiographic recording of heart activity with a commercial heart rate monitor (AliveCor Kardia). Inappropriate detection of high frequency noise led to oversensing and incorrect determination of rhythm (atrial fibrillation)—the patient is in normal sinus rhythm, with frequent PACs (premature atrial contraction—a benign cardiac rhythm abnormality).

Filters are used to suppress detection of any activity not related to QRS complexes. A high-pass amplitude filter suppresses baseline noise, frequency filters suppress non-physiological signals—both low- and high amplitude filters are commonly used. In addition, a 50 or 60 Hz notch filter is also routinely used (Figure 2).

These recording and signal classification issues affect ECG-based assessment of the cardiac cycle; which then lead to inaccuracies in HR/HRV assessment, discussed in the next sections.

## 3. Heart Rate and Heart Rate Variability

A specialized excitatory and conduction system is responsible for the regular electrical activity of the heart, which is the basis of effective, synchronized mechanical function. In healthy subjects, the sinoatrial node is the primary pacemaker of the heart. Without external factors, the intrinsic heart rate is around 100 beats per minute (as seen in a denervated, transplanted heart). As the metabolic demand of the body changes constantly, the heart rate must be regulated to maintain homeostasis [3]. The heart itself has a very limited ability to detect changes in homeostatic demands, an example of this are the stretch receptors located in the right atrium, vena cava junctions and the pulmonary veins—these regulate the heart rate via the sympathetic nervous system. Most of the regulation, however, is due to extracardiac sensors in the vasculature and end-organs, using various effector mechanisms (Table 1).

Pathologic conditions and the resulting pathophysiological processes may affect the balance of regulatory factors, with concomitant changes in heart rate. In healthy subjects at rest the parasympathetic tone is greater than the sympathetic tone, resulting in a rest heart rate in the 50–80 bpm range in most humans. Variations in HR can be observed due to cyclical (respiration, diurnal) and non-cyclical factors (postural changes, exertion, increased demand due to pathological conditions). HR and HRV thus provides a measure of the sum of factors affecting the heart.

**Figure 3:**
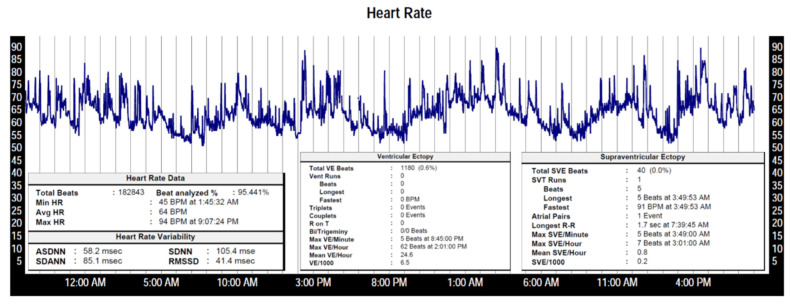
Typical graphical and textual presentation of a medical grade 48 h wearable ambulatory monitor (Holter). The heart rate trends between 45–94 bpm, with longer and shorter cycle length variations due to diurnal changes and variable activity levels. Valid data was collected during 95% of the monitoring interval, the rest was rejected due to inadequate signals. Heart rate variability parameters are automatically calculated. The percentage of ectopic, abnormal rhythms is also shown (premature atrial and ventricular contraction).

HR at rest and exercise may predict cardiovascular risk. Resting HR is an independent predictor of cardiovascular disease, stroke and sudden death [4]. HRV is a measure of variation in time between each heartbeat, representing balance between the parasympathetic and sympathetic nervous system. HR and HRV is conventionally assessed over at least a full 24 h time period, to accommodate for diurnal variation. Continuous monitoring avoids potential bias due to intermittent sampling. Due to the large amount of data recorded, most parameters are automatically calculated and then presented in a summarized format (Figure 3). It is imperative to assess the quality of the recording to assure that analysis was based on valid data.

**Figure 4:**
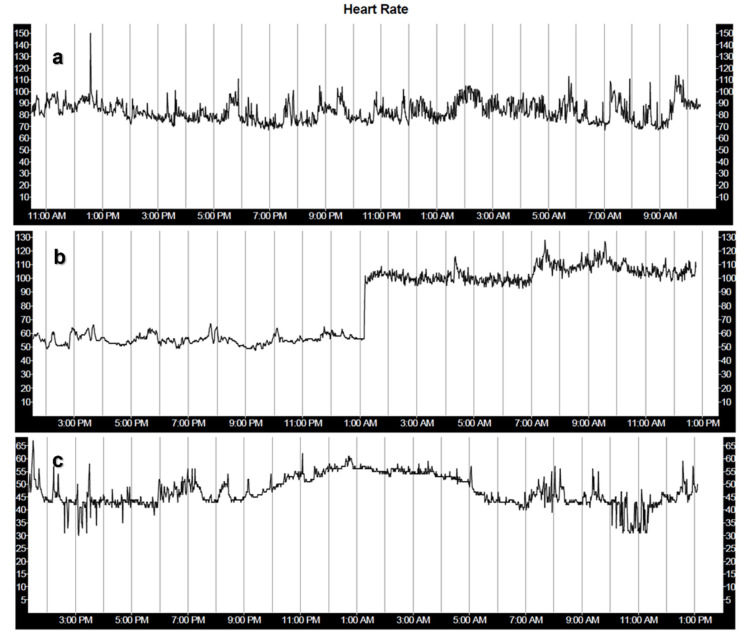
Holter monitor heart rate tracings of patients with abnormal 48 h HR/HRV. (**a**) paroxysmal supraventricular tachycardia—on the first day of monitoring, the heart rate suddenly increased to 150 bpm for a few minutes, with abrupt termination. (**b**) paroxysmal atrial fibrillation. Normal sinus rhythm in the first half of the tracing, with sudden, sustained increase in the heart rate for the second half—the arrhythmia did not terminate during the monitoring period. (**c**) sick sinus syndrome. The heart rate is low (30–65 bpm). The diurnal variation is abnormal, lower heart rate during daytime and increased heart rate at night.

Sudden changes in heart rate may correlate with pathological conditions. These may be temporary events, such as during paroxysmal supraventricular tachycardia (fast regular abnormal heart rhythm originating from the atria or atrioventricular junction). Sustained arrhythmias lead to prolonged changes in heart rate. Abnormal diurnal variations in HR may also represent pathological conditions, such as abnormal vegetative tone (parasympathetic/sympathetic imbalance), medication effects or sick sinus syndrome—loss of ability to respond to conditions requiring change in the heart rate (Figure 4).

**Figure 5:**
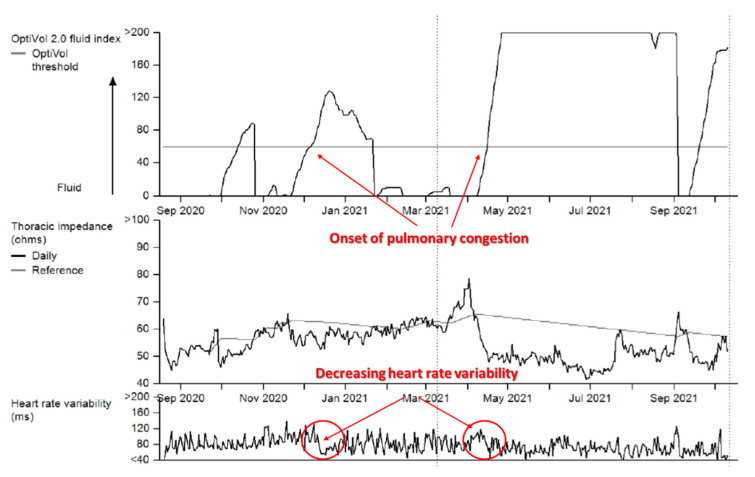
Long term heart rate variability in a patient with a Medtronic implanted biventricular defibrillator (cardiac resynchronization device implanted in patients with severe cardiomyopathy and ventricular dyssynchrony). The device continuously records heart rate and heart rate variability data. In addition, thoracic impedance is measured, which correlates with the degree of pulmonary congestion and severity of heart failure symptoms. Sudden decrease in heart rate variability may be observed at the onset of increasing pulmonary congestion (decreased thoracic impedance due to fluid buildup). As these findings may precede the onset of symptoms by several days, remote monitoring may identify high risk patients for targeted intervention.

HRV may predict adverse cardiovascular events, especially after exercise in healthy individuals, as well as in patients with heart failure with reduced cardiac contractile function [5,6]. Low heart rate variability can be caused by loss of variations of vegetive tone (such as in heart failure, due to increased sympathetic and decreased parasympathetic tone, loss of respiratory variation). Other abnormal conditions include arrhythmia, or presence of an artificial pacemaker. Longer term changes in heart rate variability may precede clinically significant events. These can be measured in patients with implanted cardiac devices, such as cardiac pacemakers, defibrillators or subcutaneous loop recorders. The changes may be difficult to detect for human interpreters and are good candidates for statistical or machine learning-based detection (Figure 5).

The most common sustained clinical arrhythmia—atrial fibrillation—has been the focus of remote monitoring recently. Early detection and treatment of this condition helps to prevent its complications, such as ischemic embolic events (stroke). In many cases AF is not symptomatic initially. It has many features that makes it an excellent target for population-wide screening: relatively high prevalence (increasing with age and in the presence of common comorbidities), ease of detection (non-invasive cardiac rhythm or HR/HRV monitoring), efficient and cost-effective treatment options. This arrhythmia is characterized by irregularly irregular RR intervals, which can be quantified by measures such as heart rate variability. Machine learning algorithms can be used for AF detection. In an ECG database analysis of 180,922 patients, the single ECG accuracy to detect AF was 79.4% (sensitivity of 79.0%, specificity of 79.5%), using a deep learning method (Convolutional Neural Network) [7]. The Cardiio Rhythm device, using non-contact photoplethysmography (assessing variations of facial skin color reflecting cardiac activity) and a Support Vector Machine algorithm, showed a 95% sensitivity and 96% specificity for discrimination of AF vs. sinus rhythm, compared to ECG in 217 patients [8].

Some HR/HRV based conditions can be easily identified by trained human interpreters as presented in this section, with proper presentation of data. More complex measures of HRV, however, require automated analysis due to the complexity of data (such as time/frequency domain or non-linear analysis, discussed in the next section).

## 4. Analysis of Heart Rate and Heart Rate Variability

Several common cardiovascular conditions affect the vegetative nervous system or lead to abnormal heart rhythms. In congestive heart failure, increased sympathetic and decreased sympathetic tone may decrease heart rate variability. Using this as a screening tool, patients at risk for lethal complications (sudden cardiac arrest due to malignant ventricular arrhythmia) may be identified and treated. Some conditions, such a heart failure exacerbation, may also be detected in the preclinical stage, where treatment is less costly and may prevent morbidity, hospitalization or mortality.

Heart rate variability may be analyzed using different metrics [9]. Time domain, frequency domain and non-linear dynamics are most used (Table 2). Time-domain metrics of HRV quantify the variability in measurements of RR intervals. Frequency-domain measurements describe the distribution of power in four frequency bands (ultra-low, ≤0.003 Hz; very low, 0.0033–0.04 Hz; low, 0.04–0.15 and high, 0.15–0.4 Hz) [10]. Vagal activity is a major component of the high frequency spectrum, while sympathetic and other factors affect the lower frequency ranges. Non-linear measures describe the unpredictability, randomness of a time series.

These measures are affected by the duration of the recording and generally assume uninterrupted data collection. The time duration of the recording affects both time and frequency domain parameters, thus their accepted normal ranges [11]. Frequently studied and analyzed intervals are daily (24 h), short-term (5 min) and ultra-short term (<5 min).

Deceleration capacity is a metric to characterize heart rhythm modulations associated with changes (deceleration or acceleration), with the goal to distinguish between parasympathetic and sympathetic influences. This technique requires the use of phase-rectified signal averaging (PRSA) and was a stronger risk predictor after myocardial infarction (area under the curve AUC 0.8), than traditional HRV parameters or even left ventricular ejection fraction (the most commonly used risk factor to estimate risk in this population, AUC 0.69) [12].

Premature ventricular contractions (PVC) are early cardiac activations originating from the ventricles, commonly due to pathological processes, however, sporadic PVCs may also be observed in healthy individuals. PVCs cause very short-term changes in heart rate (acceleration, the deceleration in healthy subjects). These can be described by metrics of heart rate turbulence (HRT) [13]. This measure has been linked to baroreflex sensitivity measures, another commonly used risk stratification method assessing autonomic abnormalities. HRT appears to be useful to predict risk post myocardial infarction, but not in non-ischemic patients [14].

## 5. Development of Ambulatory and Remote Heart Rate/Rhythm Monitoring Technology

With the growing interest in non-invasive recording and remote monitoring of biological signals and rapid technological advances beginning in the early post World War II period, ambulatory cardiac rate/rhythm monitoring became feasible since the 1950s (Table 3).

Clinicians recognized the importance of monitoring cardiac activity in non-hospitalized setting and the application of these techniques rapidly expanded thereafter [24]. The need for assisted and automated processing was also recognized early due to the large amount of data recorded even during short time periods [25]. Telemetric data transmission enabled remote monitoring even in extreme situations, such as during the early experimental and space flights at the dawn of Space Age [26].

The early automated ECG analysis algorithms used a heuristic approach, mimicking diagnostic logics used by human interpreters, with limited performance. Statistical methods of analysis, requiring processing of large amount of data, became feasible with advances in computing power. These methods are data-based and free from human interference and may theoretically surpass the accuracy what is possible by human experts [19]. Current clinical practice uses off-line interpretation with automatic or semiautomatic beat classification and rhythm analysis (in most regions, a technician is involved), final interpretation is overread by a practitioner (physician trained in interpretation of electrocardiographic data).

## 6. Sensors Used in Wearable HR/HRV Monitors

Heart rate monitoring may be pursued by direct measurement of the electrical heart activity (electrocardiography, ECG), or by measuring changes in blood flow related to cardiac activity. Most of the commercial monitoring devices are either using photo plethysmography or ECG sensors. With the miniaturization of sensors, multi-modality sensing also became feasible.

### 6.1. Photo Plethysmography (PPG)

PPG is based on measurement of changes in microvascular blood volumes. Pulses of photons are sent from an emitter which pass through the skin, reflected photons are received by a photodetector which measures variable intensity of reflected photons, which can be translated into a tachogram recording.

One of the commonly used PPG-based devices is the Apple Watch which uses green and infrared LED lights and photodiodes to detect the amount of blood flowing through the wrist. With a sampling frequency in the 0.1–1 kHz range, variations during the cardiac cycle are used to detect each systolic event, then to calculate the heart rate. The optical sensor supports a range of 30–210 bpm. The sensor can also compensate for low signal levels by increasing both LED brightness and sampling rate. The infrared sensor is used for background/baseline measurements and heart rate notifications, the green LED uses a higher sampling rate to during workout or “breathe sessions” to calculate walking average and HRV. Another commercial device, the FitBit wrist monitor uses similar LED PPG-based technology.

Simple PPG-based devices may be adequate for heart rate detection, however, validation for HRV measures is lacking (see the discussion on wearable consumer grade devices). Consumer grade devices using PPG shown good correlation of accuracy with ECG measurements. Medical grade devices using PPG have superior accuracy [27].

PPG works best when there is good contact between the device and the skin which can be challenging when used with watch and wrist band straps, especially with activity [28]. Skin color, tattoos and moisture have also been known to affect PPG accuracy [29]. A significant limitation of PPG-based heart rate measurement is the underestimation of HR in arrhythmias, especially atrial fibrillation, where early contractions generate a weaker pulse which may not be detected [30]. Despite significant advantages in terms of accuracy and ease of use, PPG based devices still have limitations.

### 6.2. ECG Based Sensors

Direct measurement of cardiac electrical activity using surface electrodes has been used in clinical practice since the 1920s. The electrode-skin interface is a major factor in signal quality. Wet Ag/AgCl electrodes are used for most clinical applications: these consist of a silver metal coated plate with an AgCl surface layer and are bathed in an electrolyte solution containing Cl−, which reduces the resistance of the last layers of the epidermis, maximizing the electrical voltage transfer between the skin and the input amplifier. These electrodes also contain an adhesive which secures the electrode in place. However, these may cause skin irritation during longer time periods.

**Figure 6:**
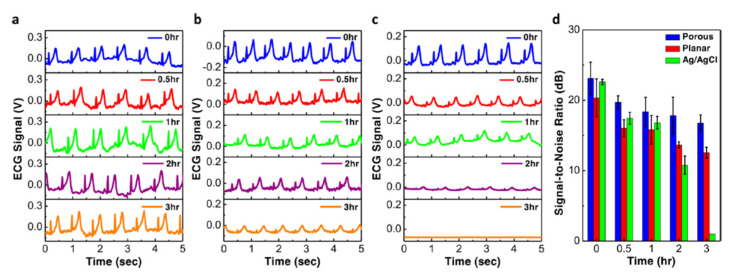
Long-term ECG signal recording. (**a**–**c**) ECG signals measured after various amount of time using (**a**) porous PEDOT:PSS/PDMS electrodes; (**b**) planar PEDOT:PSS electrodes; and (**c**) commercial Ag/AgCl electrodes. (**d**) Comparison of the signal-noise ratio between the three different types of electrodes. Maintaining a high signal-to-noise ratio over longer time periods with novel electrode technologies may help to decrease the need for frequent electrode replacements when using ECG-based wearable devices. Reprinted from [36]. No changes were made to the image, which has been published under CC BY 4.0 license (https://creativecommons.org/licenses/by/4.0/legalcode).

A newer technology—dry electrodes—does not use gel or adhesive. These electrodes are better tolerated; however, they are more susceptible to noise as the higher skin-electrode resistance requires sensor readout amplifiers with higher input impedances [31]. Materials investigated for use in dry electrodes include thin metal [32], carbon nanotube [33] and graphene [34]. A high-conductivity polymer, poly(3,4-ethylenedioxythiophene): poly(styrenesulfonate) (PEDOT:PSS), has been found to be an excellent candidate to form the basis of composite dry electrodes: it has high transmittance in the visible light spectrum (enabling transparent electrodes in wearable devices) and it is solution processable—combination with additives improves its conductivity, thermoelectric characteristics and mechanical flexibility, making it possible to optimize its characteristics for specific applications. For a wearable ECG, waterproof, long lasting (2 weeks), biodegradable (dissolving in hot water) electrodes are being designed [35]. A PEDOT:PSS composite elastomeric sponge electrode has been proposed for wearable long-term heart monitoring applications, with reduced electrode−skin contact impedance, improved signal-to-noise ratio (SNR), tolerance to motion artifacts and improved wearing tolerability. These features facilitate high quality, long term monitoring, while reducing the need for frequent electrode replacement (Figure 6) [36].

The combination of PEDOT:PSS with gelatin led to the development of a self-adherent, conformal hydrogel electrode, with less skin irritation than conventional electrodes with adhesives [37]. The ease of manufacturing and customization of biocompatible composite polymers makes them excellent candidates for further development; however, larger scale studies have not been completed yet.

Capacitive electrodes, which do not require direct skin contact and can measure ECG signals several millimeters from the skin, are under intensive investigation as these could be easily embedded in clothing or other accessories (flexible printed circuit board) [38]. These also have the potential to measure non-cardiac biological signals in ambulatory settings, such as recording brain activity via electroencephalogram.

Medical grade wearable ECG based sensors usually record more than one channel and allow for a more detailed analysis, such as QRS morphology and repolarization abnormalities to assess conduction problems, site of premature beats and ischemia. For heart rate monitoring, a single channel tracing is adequate, and this approach is used in consumer grade devices. Smart watches can record single lead ECGs with minimal patient input, where the back of the watch can act as a positive electrode and the contralateral fingertip is placed on the crown, which then acts as a negative electrode [39]. ECG sensors are still considered to be the gold standard for HR and HRV monitoring.

### 6.3. Other Sensors

Accelerometers and gyroscopes may provide additional motion information to interpret heart rate changes in context of various activities. Accelerometers primarily detect changes in linear motion in 3 axes whereas gyroscopes primarily measure angular motion. This additional input is helpful in athletic applications. In addition to motion detection, these can be used as primary sensors: data from these sensors can be fed into devices using ballistocardiogram (BCG)-based heart rate detection. BCG analyzes the repetitive motion of the human body arising from the sudden ejection of blood into the great vessels with each heartbeat [40]. The accuracy of acceleration-based sensors is affected by the site of placement on human body [41]. Integration with barometers and global positioning system (GPS) may improve the accurate assessment of activity-related HR/HRV changes—these technologies are readily available in consumer devices such as smartphones, however, this approach has not been validated yet.

Implanted devices may utilize minute ventilation, thoracic or cardiac impedance or temperature monitoring. Continuous blood pressure monitoring, or direct assessment of vegetative activity may provide useful additional information, however, these are not feasible yet in ambulatory settings. Remotely monitored implantable devices exist to intermittently measure pulmonary arterial pressure in ambulatory settings, which correlate with heart failure symptoms and may predict exacerbation (CardioMEMS) [42].

## 7. Data Processing and Analysis, Use of Machine Learning

The very first ambulatory cardiac monitors were simple wireless transmitters of signals. Local storage of data became feasible in early Holter devices using cassette recorders. Miniaturized computers later allowed local analog to digital conversion, signal processing and analysis. The explosive growth in telecommunication services most recently enabled cheap rapid transmission of large amounts of data, allowing live or near-live analysis of signals using methods with high computational needs (such as machine learning/artificial intelligence), which would not be feasible in the ambulatory monitor itself.

Data transmission can be intermittent or event-driven, such as in the case of loop recorders. This is only useful to assess correlation of events with heart activity. To assess HR trend and analyze HRV parameters, uninterrupted high-quality recording is mandatory. Data processing can be performed at set intervals or on demand.

Current consumer devices, such as the Apple Watch provide lower quality continuous heart rate measurement and higher quality on-demand measurement. Compliance with device use may also affect the data. The Apple device and service performs limited automated analysis and warns the patient to contact a medical professional if an abnormality is suspected [43]. Medical grade ambulatory monitors with continuous telemetry capabilities (such as the iRhythm Zio Patch AT) are continuously monitored by a semi-automated system with human supervision, which can alarm both the patient and the medical provider who ordered the test [44].

**Figure 7:**
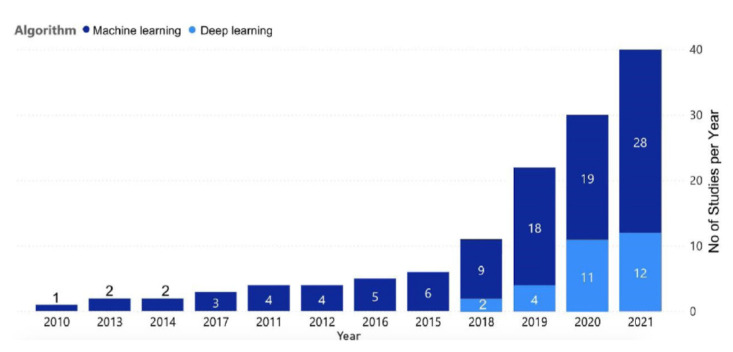
Number of HRV analysis studies using machine or deep learning within one year, from 2010 to 2021. Reprinted with permission from [23].

Machine learning is a maturing field which proved to be efficient in processing large amounts of data in many fields of medicine, including ambulatory monitoring [45]. Compared to the earlier heuristic and statistical methods, it has the potential to surpass the accuracy of expert human interpreters and to integrate multiple sensor inputs simultaneously into the decision process. Both supervised and unsupervised machine learning models can be used for heart rate variability analysis. The growing interest in machine learning-based HRV analysis is reflected by the rapidly growing number of completed studies in this field (Figure 7) [23].

In supervised learning, one or more features are selected from the dataset to train the model. The efficacy of this approach depends on the proper identification of important features, usually guided by statistics feature analysis. The algorithm discovers the feature weight and decision borders, which then allows the model to label new samples for a decision. Once the model is trained and validated, it can be used to establish specific inference results (such as establish the presence or absence of a diagnosis) on fresh data sets, without the need of further manual analysis. Supervised machine learning method, used for HRV analysis include Support Vector Machine (SVM), fuzzy Sugeno classifier (FSC), Multilayer Perceptron (MLP), Classification Additionally, Regression Tree (CART), Logistic Regression (LR), Recurrent Neural Network (RTF), Artificial Neural Network (ANN), Random Forest (RF), Gradient Boosting (GB), Decision Tree (DT), K-Nearest Neighbor (KNN), Probabilistic Neural Network (PNN), AdaBoost, Gaussian Process Classification (GPC), and Partial Least Squares Discriminant Analysis (PLS-DA) [19].

Early use of machine learning in HRV analysis was attempted for specific diagnostic applications, such as to detect obstructive sleep apnea (SVM, 93% accuracy) [46], congestive heart failure (SVM, up to 99% accuracy) [47], or diabetes mellitus (AdaBoost, DT, FSC, KNN PNN and SVM, average accuracy 90%) [48].

Deep (unsupervised) learning performs automatic labeling of the data set (such as collections of continuous heart rate recordings), without the need for prior manual feature selection—the algorithm learns patterns from the untagged data. The feature analysis is part of the algorithm and is automatically adjusted to optimize the classification results. Deep learning requires a larger learning data set to improve the model quality, compared to supervised learning. With too little training, these models are at risk for overfitting—the model is useful only in the initial data set, but not in other data sets). In HRV analysis, Convolutional Neural Network (CNN) and Recurrent Neural Network (RNN) have been used, among other less common techniques. CNN, which is based on aspects of human visual perception, was originally designed for two-dimensional data analysis (images). HRV data can be transformed into a 2D pseudoimage, then processed by CNN. Another approach is to use a CNN subclass to process the signals before they are fed to the fully connected layers of the CNN for classification. CNN, however, is not designed for temporal analysis (the 2D images only contain spatial data), which may be important in HRV analysis as some disease-defining features may not be always present in the data set. RNN avoids this limitation as it incorporates internal feedback loops, which can generate an infinite impulse response. Systems with this feature are more sensitive to temporal changes in a signal [49]. This can be further enhanced if learned parameters are used to control the memory within the algorithm, such as in Long Short-Term Memory (LSTM) networks. LSTM, used for HRV analysis was able to identify patients with moderate to severe sleep apnea with 100% sensitivity and specificity in a small study—this is a very promising screening tool as the current standard screening/diagnostic method (polysomnography) is expensive and has limited availability [50].

## 8. Consumer Grade Wearable Devices for Heart Rate and Heart Rate Monitoring

**Figure 8:**
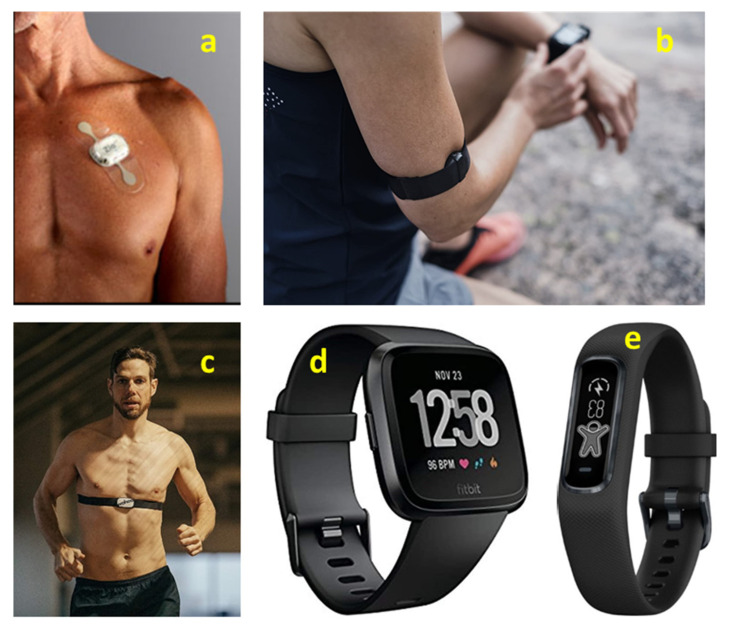
Examples of wearable cardiac monitors. (**a**) iRhythm ZIO AT chest patch monitor (medical grade, https://www.irhythmtech.com, accessed on 17 August 2022), (**b**) Polar OH1 upper arm band monitor (https://www.polar.com/us-en, accessed on 17 August 2022), (**c**) Wahoo TICKR X chest strap (https://www.wahoofitness.com, accessed on 17 August 2022), (**d**) FitBit Versa smart watch (https://www.fitbit.com/global/us/home, accessed on 17 August 2022), (**e**) Garmin VivoSmart 4 wrist strap monitor (https://www.garmin.com/en-US, accessed on 17 August 2022).

Consumer grade devices may be simple heart rate monitors, or fully ECG-based systems: smart watches/wrist bands, arm bands, chest straps or clothing devices (Figure 8). Besides HR/HRV monitoring, devices combining other sensors may also monitor other physiological parameters such as O2 saturation, respiration rate, temperature [2].

In clinical practice, patch monitors have an advantage over conventional Holter monitors due their ease of use, improved patient comfort and longer-term monitoring ime (up 2 weeks vs. up to 48 Hours). Currently the iRhythm Zio and Preventice Bodyguardian monitors are widely used, the Medtronic SeeQ MCT has been discontinued. Patch monitors require a prescription and interpretation by health professionals and are not available directly to consumers.

Upper armband monitors (Table 4) can be used for stable heart rate monitoring using PPG even during heavier physical activities. The Polar OH1 armband’s accuracy has been validated against ECG with good correlation [51]. The validation study of Everion armband monitor showed poor data compliance in a pediatric patient population [52].

Wristband monitors (Table 5) can be simple HR sensors, or smart devices with connectivity for continuous monitoring and multiple combined sensors. Despite their popularity, limited independent clinical data is available on their use. Their accuracy to detect arrhythmia is limited: the Apple Watch, Fitbit Charge HR, Garmin VivoSmart HR, and Polar A360 wrist monitors were evaluated during controlled tachycardia settings (induced during an electrophysiological study), a 15 s arrhythmia was only detected in 18.7–37.7% of the episodes. For episodes >60 s, the Apple and Polar devices had 23/23 and 19/21 episodes with at least 90% agreement between device-measured and ECG-measured HR, however, the Fitbit and Garmin devices had only 7/20 and 8/22 episodes with at least 90% agreement [53]. Using a multi-sensor analysis with the Garmin VivoSmart 4 device (accelerometry, skin conductance, skin temperature, and heart rate), self-reported stress (AUC 0.82) and craving (AUC 0.82) was classified successfully in an outpatient treatment program for a substance use disorder [54].

**Figure 9:**
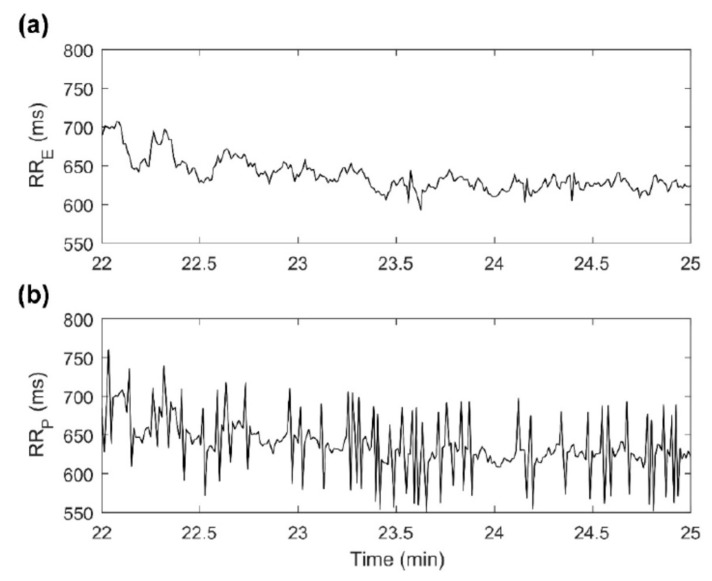
Example of RR intervals recorded by ECG and a PPG-based device (Polar H7) during recovery after exercise in one subject. (**a**) RRE = RR interval series recorded by ECG; (**b**) RRP = RR intervals series recorded by the PolarH7. Compared to the ECG, the PPG method overestimates the HRV. Reprinted from [55]. No changes were made to the image, which has been published under CC BY 4.0 license (https://creativecommons.org/licenses/by/4.0/legalcode).

Chest strap monitors (Table 6) can be conveniently used in athletic applications where limb movement would cause artifacts with peripherally placed devices. The proximity of the heart also enables ECG monitoring in these devices. The accuracy of Polar H7 was studied in 67 subjects, during and after exertion, comparing HR and HRV parameters vs. ECG. The percentage of subjects not reaching excellent agreement (concordance correlation coefficient > 0.90) was especially higher for high-frequency power of HRV and increased with exercise intensity. Unfit and older subjects with high trunk fat percentage showed the highest error in HRV measures. Although HR measures correlated well with ECG, HRV parameters showed poor concordance in 60% of subjects (Figure 9) [55].

The accuracy or HR measurement with the Zephyr Bioharness 3 devices was reviewed in a meta-analysis of 10 small trials, the correlation coefficients vs. calibrated devices were 0.74–0.99, with an agreement error of −4.81 to +3.00 beats per minute, no data exist on the reliability of HRV measures [56].

Clothing monitor (Table 7) can be integrated into a sports bra, such as the Om Bra. The device is marketed for fitness applications, but no studies have been performed to validate HR/HRV measurement accuracy. The Hexoskin smart shirt integrates multiple sensors to measure HR/HRV, respiratory, postural and activity parameters. It has been evaluated for HR applications in small studies, 4–10% difference in HR was noted, compared to ECG at various stages of exercise [57]. In elite cyclists, the degree of HR measurement error was 1.3–6.2%, affected by the level of exertion [58].

## 9. Diagnostic Uses of Ambulatory Heart Rate and Heart Rate Variability Monitoring

Risk stratification is of paramount importance in potentially fatal and preventable conditions. In many cases, the etiology is the disease process is multifactorial an no single parameter can accurately measure the risk. HR/HRV reflects the summary effect of systemic homeostatic processes and due to its relative ease of measurement, it has been studies as a risk factor since the technology became available. One of the very first uses was to monitor HR/HRV for signs fetal distress in hospitalized settings, which is now standard in labor and delivery units [59]. Power spectrum analysis of HRV can predict diabetic neuropathy in diabetes in children [60]. Frequency domain analysis of HRV allows for risk stratification for sudden cardiac death after myocardial infarction: adding measures of HRV to other risk factors identifies patients with up to 50% mortality in a 2.5-year time period, helping to identify candidates for intensive risk factor modification [10,61]. Evidence based indications guide utilization of clinical-grade ambulatory heart monitors [62] (Table 8).

We will focus on the utility of wearable devices in the next paragraphs, where applications require long term, continuous or intermittent monitoring.

### 9.1. Cardiology

Wearable devices have been utilized in many cardiovascular applications., such as early diagnosis of hypertension, atrial fibrillation detection, congestive heart failure monitoring, or prediction of sudden cardiac death due to arrhythmia.

A large number of studies have been performed recently to assess the ability of machine learning algorithms to analyze HRV in specific cardiac applications. The accuracy was modest to excellent, based on the application: arrhythmia and atrial fibrillation detection (10,000+ studied patients, DT or CNN, 95–100% accuracy) [63,64,65], long term cardiovascular risk prediction (859 patients, eXtreme Gradient Boosting, 75.3% accuracy) [66], sudden cardiac risk/ventricular fibrillation prediction (100+ patients, SVM or ANN, 67–89%) [67,68,69], hypertension detection (209 patients, RF had the best accuracy of 86%) [69]. The accuracy of HRV-based risk stratification for each indication is affected by the amount the vegetative nervous system contribution to the pathophysiology of these conditions.

Despite the good diagnostic yield of the algorithms, the large scale real-life cardiac evaluation of wearable devices so far has proven to be difficult. The Apple Heart Study was a prospective, single arm pragmatic study that has enrolled 419,093 participants. The primary objective was to measure the proportion of participants with an irregular pulse detected by the Apple Watch with AF on subsequent ambulatory ECG patch monitoring. The study was conducted virtually—screening, consent and data collection performed electronically from a smartphone app. Study visits were performed via video chat through the app, ambulatory ECG patches are mailed to the participants [70]. Multiple issues were encountered, such as data noisier than anticipated, low prevalence of the arrhythmia and poor adherence to study instructions, duplicated participant identification. The FitBit Heart Study had a similar goal: 455,699 participants enrolled by 2020, participants in whom an irregular heart rhythm was detected were invited to attend a telehealth visit and eligible participants were then mailed a one-week single lead ECG patch monitor. The primary objective was to assess the positive predictive value of an irregular heart rhythm detection for AF during the ECG patch monitor period [71]. Monitoring of healthy patients to detect arrhythmia (premature ventricular contractions) may predict ischemic risk, which can also be a target for consumer devices in the future [72].

### 9.2. Sleep Medicine

Sleep apnea is usually undetected until it is specifically suspected and evaluated due to one of its complications (atrial fibrillation, hypertension or congestive heart failure). The gold standard clinical diagnostic tool (in-lab polysomnography) is expensive, and access is limited. As sleep apnea causes marked variations in sleep pattern and vegetative balance, it is a good target for HRV-based screening methods. A sleep-staging wrist monitor (Fitbit) is comparable to polysomnography in accuracy to detect sleep phases, with 95–96% sensitivity and 58–69% specificity in detecting sleep epochs [73]. Deep learning-based methods are highly accurate in sleep apnea detection using data from pediatric and adult populations (10,000+ patients, ANN or CNN, 85–89% accuracy) [74,75]. No large scale prospective clinical studies have been completed yet.

### 9.3. Diabetes Detection and Management

One of the dreaded complications of diabetes mellitus is autonomic neuropathy, with resulting increase in cardiovascular morbidity and mortality. As the autonomous nervous system may be affected early in the disease course, HRV analysis can be used to detect diabetes or monitor the efficacy of treatment. Diabetes detection had good accuracy in small studies (200+ patients, CART or GPC, 84–93%) [76,77]. Changes in HRV detected hypoglycemia in a study of 23 patients using a wearable device (VitalConnect HealthPatch) with 55% accuracy [78].

### 9.4. Other Uses

The value or HRV analysis using wearable devices have been studied in other, pathologic and physiologic conditions. Poor correlation was found between perceived stress and HRV parameters in 657 subjects using a fitness tracker (HRV parameter predicted only 1–2.5% of variation with r^2^ 0.022–0.032) [79]. A controlled study with 60 subjects using a smart watch (FitBit) using induced stress failed to identify ubiquitous patterns of HRV and HR changes during stress [80].

HRV has also been used to guide endurance training: fitness-related beneficial remodeling of the autonomous nervous system is expected to cause measurable changes on HRV parameters, which can be used to guide the exercise plan. A meta-analysis of 8 trials (198 subjects) showed a significant medium-sized positive effect of HRV-guided training on submaximal physiological parameters (g = 0.296, 95% CI 0.031–0.562, *p* = 0.028). HRV-guided training was associated with fewer non-responders and more positive responders. The wearable monitor devices were chest straps: Polar or Omegawave, or smart watches: Polar RS800, Garmin 920XT or Ambit 2 smart watches [81].

There may be even mental health applications for wearable monitors. In 584 subjects, using a wrist monitor (Bioband) HRV analysis was performed with deep neural networks (LSTM) to classify specific mental health measures (assessed by questionnaires to specifiy cut-off points for stress, anxiety and depression). Classification accuracies were up to 83% (5 min HRV data) and 73% (2 min HRV data) [82].

## 10. Challenges

Signal acquisition and event classification (detection of each cardiac cycle) is of paramount importance, as it affects accuracy of HR/HRV calculations. The recording must be continuous, reliable, low cost and minimally burdening for the monitored subject. Newer electrodes and sensors have a great potential as they do not require adhesives and may even be woven into clothing. The small studies with consumer grade wearable devices so far validated the accuracy of HR measurements, but there is much less high-quality data supporting their use for clinically proven HRV analysis applications.

Any method that requires compliance from the subject is prone to human error. Even with medical grade loop recorders and patient education, the rate of suboptimal monitoring (failed recording or transmissions) can be as high as 45% [83]. Once further miniaturization and increased battery longevity is achieved, subcutaneous implants (like medical-grade loop recorders) with fully automated recording, data transmission and processing may improve errors related to subject noncompliance, no such consumer grade device exists yet.

Data security remains an ongoing issue. Some standardization has been achieved in medical devices, however, not in the consumer field. Consumer grade devices are protected by company protocols and follow regulations of wireless and internet-based communications. These can be quite variable based on the location. Although the data formats are proprietary, the communication protocols use worldwide available technologies and encryption methods, which are well documented and can be exploited. Data storage, transmission, signal processing and communication of results has security risks. Despite use of advanced data security systems, there is a possibility of data breaches. As these data contain sensitive information, newer tools like Blockchain have been considered to protect privacy [84]. Most of the medical grade devices are regulated by health authorities, their performance is more consistent for the most part, including data security. However, even implantable cardiac devices with remote monitoring or programming capabilities may be susceptible to cybersecurity threats and data breach [85]. In special settings, even proprietary programming and communication protocols can be hacked.

Wide scale use of cardiac remote monitoring first became available for patients with implanted arrhythmia devices, such as pacemakers and defibrillators. Remote monitoring can reduce the logistical burden for patients and caregivers, detect transient abnormalities that would be otherwise missed [86]. The technology is safe and convenient, however, there were initial concerns about the cost-efficiency due to the infrastructure and personnel needed for remote monitoring [87]. Advances in semi-automated and automated data processing kept up with the demand. This field has a great potential for further rapid advancement due to incorporation of artificial intelligence, which may surpass the accuracy of human interpreters, while keeping the costs low due to its scalability. Another important concern is integration of the data into health care data management platforms and setting reasonable expectations between patients and healthcare providers regarding frequency of data review and methods of communication. Clinical studies and simulation models confirmed the efficacy and cost-efficiency of these clinical systems: improved time-to-decision and decreased utilization of inpatient services was demonstrated [88,89,90].

Data on consumer grade devices are equivocal so far—a meta-analysis performed in 2018 analyzed the effect of wearable devices in chronic disease outcomes in adults. Wearable devices did not provide a significant benefit for health outcomes—of the 6 studies examined, one showed a significant reduction for weight loss among participants who used wearable devices; no significant reduction was discovered in cholesterol or blood pressure [91]. In contrast to clinical trials, these studies must deal with much larger populations and less organized data collection, which may lead to unanticipated challenges, such as identifying the number of unique participants, maintaining participant-level linkage of multiple complex data streams, and participant adherence and engagement [92]. Similar issues were encountered in the Apple Watch and FitBit trials.

The experience gained in these large trials will help with future designs with an emphasis on data management in digital clinical trials. With the overall growing cardiovascular disease burden, further studies addressing the efficacy and cost efficiency will be needed.

## 11. Future Directions

Interest in consumer remote heart monitor remains high and rapid growth is anticipated. Over 1000 clinical trials have been started. However, only a minority of the smart wearable devices have been cleared for clinical use by the Food and Drug Administration (13 out of 45 available devices in 2021) [1]. More data will elucidate the validity of wearable consumer grade monitor for HRV-based applications. In 2022, an estimated 67 million people planned to use a wearable device in U.S., 50% of consumers were interested in tracking their cardiac health and 68% of physicians intended to use a wearable device for patient monitoring [29]. Recent large-scale consumer studies identified issues not encountered in prior, more controlled clinical settings—these can be targeted for further investigation. As biosensor capabilities, wearable technology and automated data analytic methods continue to evolve, indications for remote monitoring may expand which will facilitate management of cardiac conditions in an efficient and cost-effective manner.

## 12. Conclusions

We have provided an overview on the physiological basis of HR/HRV, technical aspects and utility of wearable HR/HRV monitors. Although significant advances have been made in some areas in this field (new sensors and analytic methods, including machine learning), others will need further studies to validate the use of these devices in real world applications (validation of accurate measurement of specific HRV parameters, large scale prospective clinical studies for specific clinical conditions).

Even the gold standard, clinically used ECG-based methods are prone to errors in cardiac signal recording and classification, which may then lead to erroneous HR/HRV calculations—we have reviewed common examples of these issues. Novel sensor/electrode technologies with improved long-term signal-to-noise ratio are being developed, however, these have not been validated in large scale studies so far. PPG-based devices are more user-friendly compared to ECG based systems for long term monitoring, however, so far, they were consistently less accurate in HRV measurements, than devices using ECG.

The evidence for machine learning-based HR/HRV analysis is rapidly growing, however, most larger studies are from datasets and were not of prospective design. As multiple promising machine and deep learning algorithms have been identified as good candidates for HR/HRV analysis, prospective studies will help to assess their clinical and non-clinical utility. This analytic approach also has a great potential to decrease the burden on healthcare systems as most of the ambulatory data processing in clinical settings is still semi-automated and requires significant input from trained professionals.

Consumer grade devices may be advertised with indications not supported by evidence. The very large scale of ambitious studies with popular consumer-grade devices introduced unanticipated issues with potential for bias. Future research in methodology to conduct large scale, non-clinical prospective trials will help to provide high quality evidence. Data from these studies will help us to clarify the utility, limitations and pitfalls of this exciting technology.

## Figures and Tables

**Table 1:** Major regulators of the heart rate in normal conditions.

Regulatory System	Effect on HR
Autonomic nervous system	
Parasympathetic Sympathetic	Decrease Increase
Endocrine system	
Adrenal medulla Thyroid gland	Increase Increase
Intrinsic cardiac factors	
Pacemaker current in the sinoatrial node, affected by intra- and extracellular Ca^2+^/K^+^ levels	Increase or decrease

**Table 2:** Common metrics of heart rate variability (conventionally, RR or NN intervals).

Metric	Description
Time domain	
SDNN	Standard deviation of intervals
SDANN	Standard deviation of the average intervals for each 5 min segment
RMSSD	Root mean square of successive interval differences
2. Frequency domain	
Power: ULF, VLF, LF, HF	Absolute power of the ultra-low, very low, low and high-frequency bands
Peak: ULF, VLF, LF, HF	Peak frequency of the ultra-low, very low, low and high-frequency bands
LF/HF	Ratio of low-to-high frequency power
3. Non-linear	
S	Area of the ellipse which represents total heart rate variability
ApEn, SampEn	Approximate and sample entropy—regularity and complexity of a time series
DFA α1, α2	Detrended fluctuation analysis—short- and long-term fluctuations

**Table 3:** Milestones in ambulatory and remote cardiac monitoring.

Year	Technology
1949	Holter and Generelli: portable apparatus for wireless transmission of biopotential signals using 50 MHz radio waves [15]
1961	Holter: electrocardiorecorder—local storage of recorded data (Holter monitor) [16]
1960s	Holter, Ledley, Nomura: semiautomatic electrocardiogram analysis
1970s	Computer-based automated pattern recognition for ECG analysis [17]
1980s	Probability density and statistical processing of electrocardiographic data Local processing of data, real time analysis (decreased need to transmit large amount of raw data for remote analysis) [18] Improved electrode technology and signal quality—evaluation of ischemia/repolarization [19]
1990s	Digital storage, solid state memory with increased storage capacity; multi-channel and longer duration monitoring [20] Standardization of data formats [21] Implantable subcutaneous heart monitor [22]
2000s	Miniaturization of wearable and implantable devices Minimally invasive implantable monitors—Medtronic Reveal LinQ
2010s	Consumer grade remote monitoring becomes available for the general population—AliveCor Kardia (ECG), Apple Watch (heart rate)
2020s	Cloud-based monitoring services Use of machine learning/artificial intelligence for signal analysis and interpretation [23]

**Table 4:** Examples of wearable consumer grade upper arm cardiac monitors.

Device	Sensors/Parameters
Polar OH1	HR (PPG)
Everion	HR (PPG), activity, blood oxygen, temperature (more with other linked sensors)

**Table 5:** Examples of wearable consumer grade wrist cardiac monitors.

Device	Sensors/Parameters
Fitbit Luxe	HR (PPG), motion, temperature
Fitbit Versa 3	HR (PPG), temperature, GPS
Apple Watch 6	HR (PPG), ECG, motion, blood oxygen
Garmin VivoSmart HR	HR (PPG), motion
Polar A360	HR (PPG)

**Table 6:** Examples of wearable consumer grade chest strap cardiac monitors.

Device	Sensors/Parameters
Polar H7	ECG
Zephyr Bioharness 3	ECG

**Table 7:** Examples of wearable consumer grade clothing cardiac monitors.

Device	Sensors/parameters
Om Bra	HR, respiration, pedometer
Hexoskin	ECG, blood oxygen, respiration, position and acceleration

**Table 8:** Overview of common indications for heart rate/rhythm monitoring in clinical settings.

Assessment	Indication
Symptom correlation with arrhythmia	Loss or near loss of consciousness, palpitations, chest pain, shortness of breath or neurological symptoms, due to unknown cause
Risk associated with asymptomatic arrhythmia	Patients after heart attack and decreased heart function, congestive heart failure, hypertrophic cardiomyopathy
Monitor antiarrhythmic management	Rate and rhythm control assessment, proarrhythmic response detection
Pacemaker or implanted defibrillator function	Symptoms suspected due to device malfunction and not explained by device interrogation
Ischemic heart disease	Transient angina pectoris, patient unable to exercise
Atrial fibrillation	Diagnosis of atrial fibrillation, assessment of rate and rhythm control
